# Effects of Synbiotic Administration on Gut Microbiome and Fecal Bile Acids in Dogs with Chronic Hepatobiliary Disease: A Randomized Case–Control Study

**DOI:** 10.3390/vetsci11080364

**Published:** 2024-08-10

**Authors:** Verena Habermaass, Corrado Biolatti, Francesco Bartoli, Eleonora Gori, Natascia Bruni, Daniela Olivero, Veronica Marchetti

**Affiliations:** 1 Department of Veterinary Sciences, University of Pisa, Via Livornese Lato Monte, 56122 Pisa, Italy; verena.habermaass@phd.unipi.it (V.H.); veronica.marchetti@unipi.it (V.M.); 2 Department of Microbiology, Charles River Laboratories, F26D789 Ballina, Ireland; corrado.biolatti@gmail.com; 3 Department of Translational Research and New Technologies in Medicine and Surgery, University of Pisa, Via Savi 10, 56126 Pisa, Italy; francesco.bartoli@unipi.it; 4 Candioli Pharma, Via Manzoni 2, 10092 Beinasco, Italy; natascia.bruni@candioli.it; 5 Analysis Lab. BSA Scilvet, Via A. D’Aosta 7, 20129 Milan, Italy; oliverodaniela79@gmail.com

**Keywords:** canine liver disease, cholestasis, gastrointestinal signs, dysbiosis, probiotics, microbiota, chronic liver disease

## Abstract

**Simple Summary:**

Intestinal microbiome dysbiosis in human patients with chronic liver disease is known to influence disease progression. Several studies show benefits from administering probiotics/prebiotics. Similar intestinal dysbiosis was identified in dogs with chronic liver disease, but clinical trials evaluating the benefits of gut microbiome modulation in these patients are lacking. In the study, 32 dogs with chronic hepatobiliary disease were divided into two groups: one treated with a probiotics/prebiotics complex for 4–6 weeks and untreated control group. All dogs underwent clinical evaluation, complete anamnesis, bloodwork, abdominal ultrasound, fecal bile acids, and gut microbiome evaluation. Treated dogs showed a significant reduction in biochemical markers of liver injury and resolution of gastrointestinal signs compared to the control group. Some qualitative modifications of the gut microbiome were observed, but no significant changes in the fecal bile acids profile were detected in association with the probiotics/prebiotics administration.

**Abstract:**

Alteration in the gut microbiome in human patients with chronic liver disease is a well-known pathophysiological mechanism. Therefore, it represents both a diagnostic and therapeutical target. Intestinal dysbiosis has also been identified in dogs with chronic liver disease, but clinical trials evaluating the effectiveness of synbiotic administration are lacking. Thirty-two dogs with chronic hepatobiliary disease were equally randomized into two groups: one treated with a synbiotic complex for 4–6 weeks (TG) and one untreated control group (CG). All dogs underwent clinical evaluation, complete anamnesis, bloodwork, abdominal ultrasound, fecal bile acids, and gut microbiome evaluation at T0–T1 (after 4–6 weeks). Treated dogs showed a significant reduction in ALT activity (*p* = 0.007) and clinical resolution of gastrointestinal signs (*p* = 0.026) compared to control dogs. The synbiotic treatment resulted in a lower increase in *Enterobacteriaceae* and *Lachnospiraceae* compared to the control group but did not affect the overall richness and number of bacterial species. No significant changes in fecal bile acids profile were detected with synbiotic administration. Further studies are needed to better evaluate the effectiveness of synbiotic administration in these patients and the metabolic pathways involved in determining the clinical and biochemical improvement.

## 1. Introduction

The gut microbiome (GM) encompasses the total amount of bacteria, archaea, fungi, and viruses of the gastrointestinal tract, contributing to various intestinal and extraintestinal functions [1]. The metabolism of the gut microbiome relies on a wide range of molecules, including primary and secondary bile acids (BAs) [2]. In human medicine, research on GM modifications is increasing, particularly concerning liver diseases, where multiple mechanisms may be involved. For this reason, disturbances of the gut–liver axis can potentially play a key role in several liver diseases. Perhaps due to the connection between BAs and microbiota, GM dysbiosis has been studied and documented in cholestatic biliary disorders, specifically in primary biliary cholangitis and primary sclerosing cholangitis [3,4]. Primary BAs are synthesized from cholesterol mainly by the liver and later secreted into the small intestine through bile. Most primary BAs (90%) undergo active ileal reabsorption, returning to the liver via the so-called enterohepatic circulation. The small amount of primary BAs remaining in the gastrointestinal tract is either dehydroxylated by GM into secondary BAs or deconjugated and passively absorbed in the colon [5]. BAs serve several functions: acting as emulsifiers, promoting the absorption of lipids (including fat-soluble compounds and vitamins) and steroid hormone-like molecules, and they regulate glucose, lipids, and energy metabolism. The GM plays a central role in the conversion of primary BAs to secondary BAs, the reabsorption of primary bile acids, and bile acid homeostasis. Conversely, bile composition and its flow can influence GM [6]. In human patients with chronic hepatobiliary disease, modulating the GM may improve their outcomes [7,8,9].

Research on the canine GM is expanding, with a primary focus on intestinal disorders. In dogs with chronic enteropathy, dysbiosis characterized by a reduction of *Clostridium hiranonis* is associated with an altered primary/secondary BAs ratio, where primary BAs increase and secondary BAs decrease [10].

A recent study investigated the potential interaction between liver disease and the GM in dogs [11], concluding that dysbiosis is common in dogs medically managed for CPSS, although the clinical significance is unclear [11]. Another recent study highlighted the presence of GM dysbiosis in a group of 65 dogs with various chronic hepatobiliary diseases (CHBDs), particularly in relation to biliary cholestasis [12].

Given these premises, the aim of this prospective case–control study was to evaluate differences in clinical signs, biochemical parameters, intestinal GM, and fecal bile acid compositions in dogs with CHBD, in relation to the administration of a commercial synbiotic complex and its potential benefits in terms of clinical and biochemical improvement.

## 2. Materials and Methods

The study was conducted in accordance with the Declaration of Helsinki and approved by the Ethics Committee of the University of Pisa (protocol code n. 41, date of approval: 29 October 2020).

Client-owned dogs referred to the Internal Medicine Service of the Veterinary Teaching Hospital “Mario Modenato” at the University of Pisa between January 2021 and January 2022, with a diagnosis of chronic hepatobiliary disease (CHBD), were included in the study. The CHBD diagnosis was based on medical history, physical examination, hematology, blood biochemistry, and abdominal ultrasonography.

To be included in the study population, dogs had to present with at least two persistently elevated (>2 months) liver enzymes among Alkaline Phosphatase (ALKP) (45–250 U/L), Gamma-Glutamyl Transferase (GGT) (2–11 U/L), Aspartate Aminotransferase (AST) (15–40 U/L), or Alanine Aminotransferase (ALT) (20–70 U/L). Additionally, they needed to show concurrent ultrasonographic hepatobiliary alterations suggestive of chronic hepatobiliary disease (such as hepatic parenchymal alterations, biliary sludge, mobility of the biliary sludge, cholelithiasis, gallbladder wall thickening, intrahepatic biliary tree dilatation, mineralization of the intrahepatic biliary tree, and common biliary duct dilatation). Degenerative hepatopathy was diagnosed based on ultrasonographic features suggestive of a degenerative pattern (enlarged/hyperechoic liver) and liver cytology compatible with lipid accumulation (lipidosis). Chronic cholestatic hepatobiliary disease was diagnosed if ultrasonographic features indicated biliary tract disease (reduced motility of the biliary sludge, cholelithiasis, gallbladder wall thickness, intrahepatic biliary tree dilatation, mineralization of the intrahepatic biliary tree, and common biliary duct dilatation) and if there were at least two overrange parameters among ALKP, GGT, total bilirubin, cholesterol, and whether chronic hepatitis was histologically assessed. Information on hepatic histological diagnosis was collected when available. Dogs were excluded from the study if they had a history of probiotic/prebiotic/synbiotic or antibiotic administration two months prior to the visit, or if they had a previous diagnosis of primary chronic enteropathy and/or significative ultrasonographic signs of chronic enteropathy or pancreopathy. Additionally, dogs with ongoing acute hepatobiliary disorders were excluded from the study population.

At the time of inclusion (T0), information on diet and chronic gastrointestinal signs (persisting or recurrent diarrhea and/or vomiting) was collected. Clinical examination was conducted, along with complete blood count and biochemical profile (including total proteins, albumin, ALKP, AST, GGT, ALT, total bilirubin, cholesterol, urea, creatinine, DGGR lipase, electrolytes, and minerals) after >12 h of fasting. A fresh fecal sample, collected by the owner in a sterile container, was immediately divided into three aliquots. One aliquot was used for a copro-parasitologic exam (saline flotation) to exclude the presence of gastrointestinal parasites. The remaining two aliquots were subsequently frozen at −20 °C (within 1h of collection) and at −80 °C within 48 h.

Prior to the start of the study, 32 dogs were randomly assigned to one of two experimental groups, each consisting of 16 dogs, using a prior computer-generated list. The treatment group was treated with the synbiotic complex Florentero^®^ Candioli (TG), while the control group did not receive the synbiotic (CG). Florentero^®^ was administered according to the company’s recommendation based on dogs’ body weight (1 tablet/10 Kg body weight). The technical sheet and composition of the administered product are reported in Table 1. Neither diet nor ongoing therapies (i.e., hepatoprotectants) were altered during the clinical trial to avoid bias.

After 4–6 weeks (T1), the dogs underwent a routine clinical check, including an evaluation of gastrointestinal signs and bloodwork. A second fecal sample was collected using the same methods applied at inclusion.

At the end of the study, frozen-stored fecal samples were sent to the external laboratories for GM analysis (Analysis Lab Labospace, Milan, Italy) and fecal bile acids measurement (Department of Translational Research and New Technologies in Medicine and Surgery, University of Pisa, Pisa, Italy). After analysis, microbial taxa and primary and secondary fecal bile acids were collected and analyzed between TG and CG at the T0 and T1 timepoints. Complete resolution of clinical signs (diarrhea and/or vomiting) and improvement in ALT levels, defined as a ≥30% reduction in the T0 value, were investigated at T1. Gastrointestinal improvement was defined as the complete resolution of clinical signs (vomiting, diarrhea).

Stool samples consisting of 4 gr fresh fecal material were stored at −80 °C until DNA extraction. Bacterial DNA was extracted from the frozen stool samples with the Stool Cell DNA Extraction Kit (TANBead, W6SCS66, Taiwan Advanced Nanotech Inc., Taiwan) on the Maelstrom 9600 TANBead automated extraction platform, following the manufacturer’s instructions. The extracted DNA (6 ng for each sample) was used for microbiome analysis with the NGS method on the IonTorrent platform (Thermo Fisher Scientific, Waltham, MA, USA). The Ion 16S™ Metagenomics Kit (Thermo Scientific™, Waltham, MA, USA), which analyzes 7 of the 9 hypervariable regions of the bacterial 16S rRNA, was used for library construction. Libraries were sequenced on the Ion GeneStudio™ S5 System (Ion Torrent™, Thermo Fisher Scientific, Waltham, MA, USA). Before analysis, sequences (or reads) obtained from sequencing were cleaned using dedicated algorithms to remove short and low-quality reads. Sequences shorter than 10 base pairs were excluded. Sequences generated were directly analyzed using the Ion 16S™ metagenomics analysis module within the Ion Reporter™ v. 5.20.2.0 software, utilizing the premium curated Applied Biosystems™ MicroSEQ™ ID 16S rRNA and curated Greengenes databases, enabling a semiquantitative assessment of complex microbial samples. The raw sequence data were analyzed using MicroBACT Software (Oxoid™ Microbact™ Software Program 070722Awhich performs taxonomic assignment by aligning individual reads with the RDP database (Ribosomal Database Project). Only sequences meeting certain alignment criteria are associated by the analysis system at the taxonomic level of the species (evaluation of the minimum length of the sequence that aligns with the reference sequence and percentage of similarity). Sequences were processed and analyzed with QIIME 2 (Quantitative Insights into Microbial Ecology 2) v. 2019.7. DADA2 was used to create the amplicon sequence variant (OTUs) table after demultiplexing the sequences. Alpha diversity was evaluated with QIIME2 using Chao1 (richness), Shannon diversity, and observed ASVs metrics. Beta diversity was estimated using Bray–Curtis, Jaccard, and unweighted UniFrac distance matrices, with plots generated in QIIME2.

Chenodeoxycholic acid (CDCA) and Cholic acid (CA) were evaluated as primary bile acids, while Ursodeoxycholic acid (UDCA), Deoxycholic acid (DCA), and Lithocholic acid (LCA) were evaluated as secondary bile acids.

A dry amount of 5–10 mg of lyophilized feces was combined with sodium acetate buffer (100 mM, pH 5.6; 250 µL) containing 15 units of cholylglycine hydrolase and 150 units of sulfatase. The mixture was incubated at 37 °C for 16 h. To stop the reaction, isopropanol (500 µL) and 1 N NaOH (100 µL) were added, and the mixture was heated at 60 °C for 120 min. Afterward, an internal standard (IS) of 50 nmol of norDCA and 0.1 N NaOH (3 mL) was added. Bile acids were extracted from the fecal matrix by ultrasonication in an ultrasonic bath at room temperature for 1 h. After centrifugation, the supernatant was transferred to a glass test tube, and the pellet was washed with 0.1 N NaOH (2 mL). The combined extracts were applied to a Waters Sep Pak tC18 cartridge (500 mg sorbent), primed with methanol (10 mL) and water (10 mL). The cartridge was washed sequentially with water (1 × 5 mL), 15% acetone (1 × 4 mL), and another 5 mL of water (1 × 5 mL). Retained fecal bile acids were eluted with methanol (1 × 6 mL) and evaporated to dryness under a nitrogen stream below 40 °C.

The extracted unconjugated bile acids from either method were derivatized to their 24-phenacyl esters. To the dried extract, 10 mg/mL of TEA in acetone (150 µL) and 12 mg/mL of phenacyl bromide (2-acetobromophenone) in acetone (150 µL) were added. The mixture was heated at 50 °C with ultrasonication in a screw-capped glass tube for 1.5 h. The reaction mixture was then diluted with acetone (2 mL) and applied to a Waters Sep-Pak^®^ (Waters®, Framingham, MA, USA) silica cartridge (500 mg sorbent) primed with acetone (5 mL). The column was eluted with acetone (4 mL) to completely elute the bile acid 24-phenacyl esters, and the collected effluent was dried under a nitrogen stream.

The obtained residue was resuspended in 82% methanol (200 µL), filtered through a 0.45 µm filter, and an aliquot (20 µL) was injected into the HPLC instrument. The set up included a Jasco separation module equipped with a DAD detector, controlled by chrome nav software (ChromNAV 2.0 HPLC Software). A Phenomenex Luna C18 column (Phenomenex Inc., Bologna, Italy), 1500 mm × 3 mm inner diameter, particle size 3 µm, fitted with a guard column (20 mm × 3 mm id) was used for the separation, maintained at 40 °C during the analysis. The mobile phase was methanol (82%), with a constant flow rate of 0.7 mL/min. Individual bile acid 24-phenacyl esters were detected by monitoring their absorption at 254 nm [13].

Unless otherwise specified, statistical analyses were conducted using R software, version 4.3.2.

For microbial taxa, the phylum was expressed as a percentage of the total microbial population, while family, genus, and species were expressed as OTUs (operational taxonomic units).

Eventual differences in biochemical parameters between TG and CG at T0 were investigated through the Mann–Whitney U test, followed by the Kolmogorov–Smirnov test to evaluate distribution. 

Particular attention was given to serum ALT, since it is currently considered a reliable marker of chronic liver injury [14]. The synbiotic influence on the gastrointestinal signs’ resolution and ALT reduction was evaluated via the Fisher–Freeman–Halton exact test, conducted with IBM SPSS version 29.0.1.0. ALT reduction was defined as a decrease of at least 30% from T1 compared to T0.

Alpha diversity measures the diversity within a single microbial community. It measures both richness (number of different species or taxa) and evenness (the relative abundance of those species or taxa) of microorganisms within a specific environment. Essentially, it provides an indication of the number of different types of microorganisms present in a particular sample and how evenly they are distributed [15]. The alpha diversity of the gut microbiome was assessed using Shannon’s diversity index (reflecting both richness and evenness). To assess the correlation between the increase in alpha diversity and gastrointestinal improvements or ALT reduction, a point-biserial correlation was performed. An increase in alpha diversity was defined as the value of alpha diversity at T1 divided by the value of alpha diversity at T0.

To evaluate the correlation between alpha diversity and species number, a Pearson’s product-moment correlation was performed. The values used for both variables were those measured at T0, i.e., pre-treatment.

Linear mixed-effects models were used to compare species number and alpha diversity values between T0 and T1 for both TG and CG. The models included random intercepts for each dog to account for within-subject correlation.

We investigated the association between synbiotic consumption and changes in gut microbiome composition using logistic regression. Dogs were categorized based on whether they received the synbiotic (coded as 1) or not (coded as 0). The independent variable was the change in bacterial abundance (T1–T0) for 24 families, measured between an initial time point (T0, synbiotic administration) and a subsequent measurement (T1). The dependent variable was synbiotic consumption. Families with a statistically significant association with synbiotic consumption were identified using stepwise variable selection. The variables obtained from this stepwise procedure were further filtered via backwards selection using the Chi-squared test. Variables with a *p*-value < 0.001 were selected. The families identified in this way were used to produce linear regression models. In these models, the dependent variable was the change in bacterial abundance of the family, while the independent variable was synbiotic consumption, coded binarily as in the logistic regression model. To estimate the change in bacterial abundance for the dogs that did not receive the synbiotic, the intercept of the linear regression was calculated. 

To estimate the change in bacterial abundance for the dogs that received the synbiotic, relative to the same change in the control group, the slope coefficient of the linear regression was calculated.

To investigate changes in fecal bile acids between T0 and T1 in both groups, according to non-normal distribution assessed through the Kolmogorov–Smirnov test, the Mann–Whitney U test was applied.

To assess the correlation between *C. hiranonis* and BAs, a Pearson’s product-moment correlation was performed. The increase in *C. hiranonis* over time (T1–T0) was compared with the increase in primary BAs over time. This comparison was repeated with the same species for secondary BAs and for the primary BAs/secondary BAs ratio.

For fecal bile acids, linear mixed-effects models were fitted to compare values between T0 and T1 in TG and CG. The models included random intercepts for each dog to account for within-subject correlation.

## 3. Results

### 3.1. Animals

In this prospective randomized study, 32 client-owned dogs with CHBD were enrolled. They were randomly assigned to two groups: TG, which had 15 dogs, and CG, which had 16 dogs. One dog in the TG group was excluded from the study, after the owner decided to discontinue the treatment.

Population signalment, diagnosis, serum ALT, and gastrointestinal signs at T0 and T1 are reported in Table 2. Serum biochemical findings of TG and CG at T0 are detailed in Table 3, with no significant differences between TG and CG for the biochemical parameters considered.

In TG, 6 out of 11 dogs (55%) presented clinical signs (vomiting and/or diarrhea) at T0 and only 1 dog still had gastrointestinal clinical signs at T1. In CG, 4/16 (25%) patients had clinical signs at T0. By T1, three of these dogs presented a clinical resolution, whereas four dogs presented clinical signs, including three that were asymptomatic at T0.

**Figure 1:**
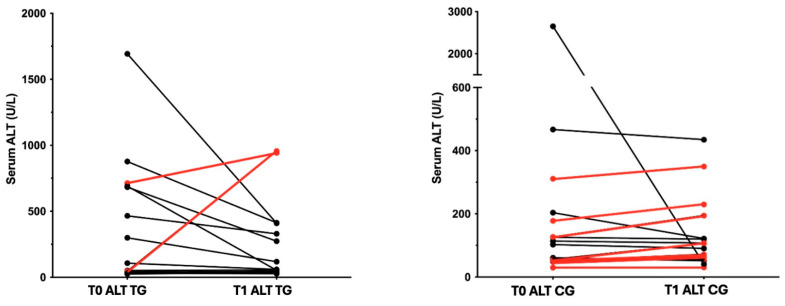
Trend of serum ALT (U/L) in TG (treatment group) and CG (control group) between T0 and T1 timepoints. Reference range 20–70 U/L. Black lines refers to a reduced ALT concentration between T0-T1, red lines refers to its increase.

According to serum ALT, 8/15 dogs (53%) in the TG group and 9/16 dogs (56%) in the CG groups presented increased ALT at T0. As reported in Figure 1, 7/8 patients (87.5%) in the TG group showed an improvement in serum ALT, while 1 patient presented increasing ALT from a normal to abnormal level at T1. In the CG group, only 2/8 patients (25%) experienced an improvement in serum ALT, and 2 patients experienced an increase in ALT from normal to abnormal levels.

Synbiotic consumption was associated with both the resolution of gastrointestinal clinical signs and a reduction in ALT (*p*-value = 0.026 and *p*-value = 0.007, respectively).

### 3.2. Microbiome

**Figure 2:**
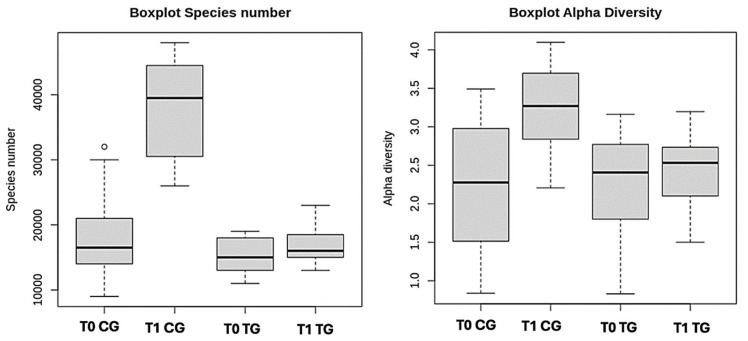
Boxplot of the alpha diversity and species number (OTU) of the CG (control group) and TG (treatment group).

A moderate correlation between alpha diversity and species number was observed (correlation value: 0.53, 95%CI = 0.22–0.75, *p*-value = 0.002). No correlation was found between an increase in alpha diversity and improvement in gastrointestinal signs (*p*-value = 0.55). The alpha diversity and species number at T0 and T1 for each group are reported in Table 4 and Figure 2.

The main difference between the test and the control group is the change in species number richness and species evenness over time. Specifically, the number of species and the alpha diversity increased over time in the control group (*p*-values < 0.001), while no significant change was observed in the treatment group (*p*-values > 0.2).

**Figure 3:**
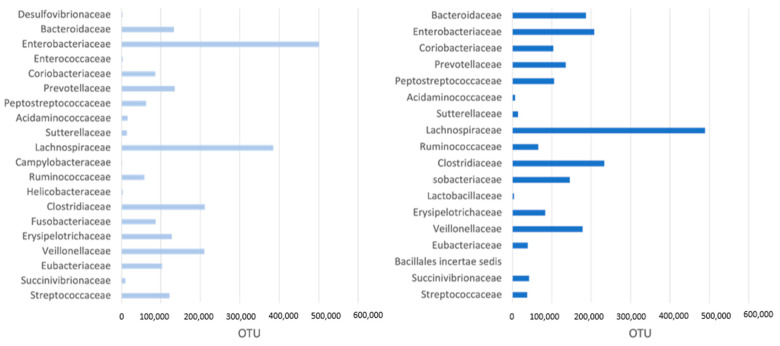
Most represented bacterial families (>1000 OTU) in the fecal microbiome of TG (treatment group) at T0 (**left**) and T1 (**right**).

**Figure 4:**
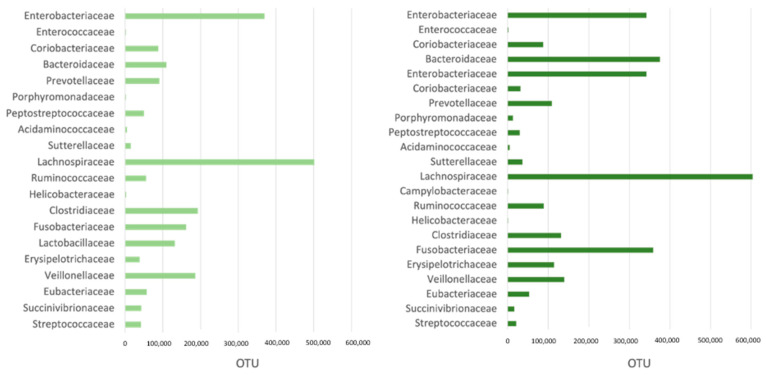
Most represented bacterial families (>1000 OTU) in the fecal microbiome of CG (control group) at T0 (**left**) and T1 (**right**).

Fecal microbiome assessment showed as the most represented families the Bacteroidaceae, Clostridiaceae, Enterobacreriaceae, Fusobacteriaceae, Lachnosporiaceae, Peptostreptococcaceae, Prevotellaceae, Ruminococcaceae, Streptococcaceae, and Veillonellaceae. Bacterial families at T0 and T1 in both TG and CG are reported in Figure 3 and Figure 4.

The *Enterobacteriaceae* and *Lachnospiraceae* families were significantly associated with synbiotic administration (*p*-values = 0.0188 and 0.0130, respectively). Dogs not receiving synbiotic exhibited an increase over time, i.e., from T0 to T1, of 23,112 units (95% CI: 749–45,476. *p*-value < 0.05) in *Enterobacteriaceae* and of 37,730 units (95% CI: 23,007–52,454. *p*-value < 0.0001) in *Lachnospiraceae*. 

For dogs receiving the probiotic, *Enterobacteriaceae* increased by 42,660 fewer units (95% CI: 10,511–74,810. *p*-value < 0.05) relative to the control group, and it increased in *Lachnospiracae* by 31,892 fewer units (95% CI: 10,727–53,059. *p*-value < 0.01) relative to the control group. Overall, these data suggest that the dogs treated with probiotics experienced a lower increase in *Enterobacteriaceae* and *Lachnospiraceae* relative to the control group.

### 3.3. Fecal Bile Acids

The single fecal bile acids, primary, secondary, and primary/secondary BAs ratio did not show statistically significant differences between T0 and T1 in either the TG or CG groups. Results are shown in Table 5. 

No correlation was found between an increase in *C. hiranonis* and the increase in primary BAs, secondary Bas, or primary/secondary BAs ratio (*p*-values > 0.35).

## 4. Discussion

Many studies in veterinary medicine focus on the potential effects of probiotics/prebiotics administration on both healthy [16,17,18] and chronic enteropathy dogs [19]. However, there is limited literature on the potential effects on dogs with chronic hepatobiliary disease [12]. The role of GM in the pathogenesis of various human liver diseases is well known, with evidence of dysbiosis contributing to its establishment and progression [20]. The gut–liver axis and potential role of dysbiosis in the development of nonalcoholic fatty liver disease (NALFD) and nonalcoholic steatohepatitis have been studied in mice and humans [21,22,23]. These studies have identified several mechanisms of this link, including modifications in the metabolism of short chain fatty-acids (SCFAs) [21], increased intestinal permeability, and LPS-driven activation of Toll-like receptors and inflammasomes [22,23]. Many clinical trials involving probiotics, prebiotics, and synbiotic administration in humans and rodents with chronic liver disease have shown positive effects on disease clinical activity and metabolism [24,25,26]. A recent study conducted on healthy dogs points out that a 28-day administration of a probiotic strain of *Enterococcus faecium* did not result in any significant changes in ALT throughout the clinical trial [27]. Our results showed that synbiotic administration was associated with both gastrointestinal improvement and ALT reduction, although it did not influence either the alpha diversity or the number of species. Neither diet nor other ongoing treatments were modified between T0 and T1, aside from the synbiotic administration in the treatment group. Thus, it is more likely that the observed changes could be related to its action. This suggests that the positive effects of synbiotics on the liver may involve multiple mechanisms, which could be partially independent of the richness of the microbiome. Even in healthy humans, changes in the GM that do not affect the overall number of species or alpha diversity have been reported after the administration of multi-strain probiotics [28] or increased dietary fiber quota [29,30], despite a reduction in disease activity. This may be explained by the fact that specific types of dietary fiber can enhance and involve the metabolism and activity of certain bacterial strains more than others (i.e., nonstarch-polysaccharides involve *Bifidobacterium*, *Feacalibacterium*, *Ruminococcus*, and *Lactobacillus*, while resistant/non-digestible oligosaccharides involve *Bifidobacterium*, *Lactobacillus*, and *Akkermania*) [31]. Thus, according to our results, the administration of both probiotics and prebiotics could affect the qualitative composition of the microbiome without directly altering its evenness and richness. From this perspective, it is possible that intestinal metabolomics may be modulated by the administration of a synbiotic complex without significantly impacting GM alpha diversity and evenness.

Dysbiosis has been identified in dogs with chronic hepatobiliary disease [12]; however, there is limited literature on the use of pre/pro/synbiotics in dogs with these specific conditions and the associated GM modifications. Although not statistically significant, the treatment group showed an increase in *Fusobacteriaceae* and *Bacteroidaceae* unlike the control dogs. These taxa are involved in SCFA metabolism [32,33] and have been reported to be reduced in dogs with cholestatic chronic liver disease [12] and with chronic enteropathy [34]. SCFAs, which derive from bacterial metabolism, have numerous positive effects on the gastrointestinal tract, including the maintenance of the intestinal epithelial barrier functions and a significant immunomodulatory activity [35]. 

Furthermore, dogs treated with synbiotic experienced a significantly lower increase in *Enterobacteriaceae* and *Lachnospiraceae* relative to the control group. In humans, NAFLD has been associated with an increased number of Gram-negative bacterial species, including endotoxins and LPS-producers *Enterobacteriaceae* [36,37,38]. In addition, in NAFLD, the activation of innate immunity seems to be associated with its development [39]. Specifically, gut-derived toxins are thought to play a causative role in liver inflammation, particularly in the onset and progression of chronic liver diseases [38]. In fact, the LPS-binding protein complex can activate Toll-like receptors, triggering an inflammatory cascade that contributes to the progression of NAFLD [40,41].

The role of *Lachnospiraceae* in the human gut is controversial, as their numbers can increase in subjects with various diseases. However, they have shown positive effects producing beneficial metabolites for the host, as SCFAs (hydrolyzing starch and other sugars to produce butyrate [42,43,44]). It is important to highlight that *Lachnospiraceae, Blautia* in particular, play a central role in undigested carbohydrate metabolism [45]. Despite the beneficial effects of SCFAs [46], the carbohydrate digestion by the GI microbiota can affect blood glucose levels. In fact, a high abundance of *Lachnospiraceae* has been positively correlated with glucose and/or lipid metabolism, indicating a possible metabolic disturbance [47,48,49]. Indeed, an association between *Lachnospiraceae* and type 2 diabetes has been observed in both humans and mouse models [50,51]. Patients with NAFLD, NASH, or significant fibrosis exhibited a greater abundance of *Lachnospiraceae* in their GM [52]. Additionally, *Lachnospiraceae* were significantly increased in human patients with primary sclerosing cholangitis together with inflammatory bowel disease (IBD) compared to healthy controls and patients with IBD alone [53,54].

No significant differences were found in fecal bile acids (BAs) between TG and CG when comparing T0 and T1 levels. In humans, primary BAs are essential for various physiological processes in the gut but can also exert negative effects under certain conditions, such as promoting inflammation [55], disruption of intestinal barrier function [56], dysregulation of GM [57], and colon carcinogenesis [58]. On the other hand, secondary BAs, which are formed through the microbial metabolism of primary BAs in the colon, can promote various positive effects on gut health. They exhibit anti-inflammatory properties [57], help regulate gut barrier function [59], modulate the GM [60], and may have potential anti-cancer effects [58]. Dogs with chronic enteropathy are known to have increased primary BAs and decreased secondary BAs [34]. However, our results are not consistent with this finding. It is possible that the metabolism and pathophysiology of these compounds differ between dogs with chronic enteropathy and those with chronic liver disease. The metabolism of BAs in chronic liver disease and cholestatic liver disease is complex, as it involves both liver function impairment and eventual cholestatic processes. During cholestasis, a decrease in BA synthesis serves as a protective mechanism against hepatic BA accumulation and cytotoxicity [61,62]. Our population exhibited a high prevalence of biliary involvement which could be commonly associated with cholestasis. Therefore, we can hypothesize that bile flow, bile composition, and the fecal bile acid pool can differ between dogs with different diseases and healthy dogs. Despite the lack of statistical significance, dogs treated with the synbiotic complex showed a tendential increase in secondary fecal BAs compared to control group, which showed a slight decrease. Further studies with a larger cohort of dogs with chronic liver disease at various stages, along with clinical/biochemical signs of liver failure, are needed to better evaluate changes in fecal bile acids. Moreover, in the authors’ opinion, considering the high interindividual variability in the microbiome and associated metabolomic factors, individual monitoring could provide relevant elements.

Levels of *C. hiranonis* do not appear to be associated with fecal BA concentrations. This may be because multiple bacteria are involved in BA metabolism. In humans, the GM plays a crucial role in bile acid metabolism, converting primary bile acids into secondary bile acids through various enzymatic reactions. Key bacterial species involved in this process include, for example, *Clostridium scindens* [57], *Clostridium hiranonis* [63], *Bacteroides* spp. [64], and *Eubacterium* spp. [65].

This study should be considered in light of its limitations. A control group of healthy dogs or a placebo group was not included, and dogs with end-stage liver disease were not represented in the population. Another limitation is the inclusion of a heterogeneous population of dogs with different types of CHBD, which may involve varying physiopathogenetical mechanisms. Additionally, hepatic histological assessments were not performed for each dog, preventing definitive characterization of CHBD for each case based on individual therapeutical and diagnostic processes. Although no changes were made to diet or treatment during the study, a standardized pre-study diet was not applied to the study population. Moreover, given the chronic nature of the disease, including a long-term timepoint with extended treatment duration could have been associated with more pronounced changes in the gut microbiome and fecal bile acids profile. We may suppose that a longer treatment could be necessary to modulate a GM that is consistently and chronically altered. As a future perspective, it would be beneficial to include a larger cohort of CHBD dogs with different kinds of hepatopathies and biochemical patterns. This would allow for a comparative assessment of synbiotic effectiveness across different conditions.

## 5. Conclusions

Dogs with chronic hepatobiliary disease treated with a synbiotic complex for 4–6 weeks showed a significant reduction in ALT activity as well as significant clinical improvement in gastrointestinal signs compared to untreated control dogs. While some qualitative modifications of the gut microbiome were observed, these did not affect the overall richness and number of species. No significant changes in fecal bile acids profile were detected in association with the synbiotic administration.

These results are preliminary, and further studies with larger cohorts, along with metabolomics assessments, are needed to more comprehensively evaluate the effectiveness of synbiotic administration in these patients. Such studies should also explore the metabolic pathways involved in determining the clinical and biochemical improvement found.

## Figures and Tables

**Table 1:** Composition of the synbiotic product employed in the study (Florentero^®^ Candioli, Italy).

Type of Addictive (per Kg)	Component
Vitamins	- Niacinamide 3a315 19.200 mg - Vitamin B1 3a820 2.500 mg - Vitamin B6 3a831 800 mg - Vitamin B2 3a825ii 640 mg - Vitamin E 3a700 8 Ul
Microbial component and yeast	- Enterococcus faecium DSM 10663/NCIMB 10,415 4b1707 2.8 × 10^12^ CFU - Lactobacillus acidophilus CECT 4529 461,715 8.6 × 10^12^ CFU - Yeast products (Saccharomyces Cerevisiae DSM 34246)
Organoleptic additives	- *Vaccinium myrtillus* L.: CoE 469 6.720 mg - *Thymus vulgaris* L.: CoE 456 3.960 mg - *Camellia sinensis* (L.) O. Kuntze: CoE 451 1.840 mg - Product by apple (all parts)
Other	- Stabilizers (Microcrystalline cellulose) - Emulsifiers (Lecithins) - Anticaking agent (Colloidal silica) - Mineral salt (sodium, potassium)

**Table 2:** Demographic distribution of canine population, serum ALT (U/L) and presence of gastrointestinal signs (diarrhea, vomiting) at T0 and T1 considering TG (treatment group) and CG (control group) at T0.

Demographics						T0		T1	
N	Group	Breed	Sex	Age (y)	Diagnosis	ALT	GI Signs	ALT	GI Signs
**1**	TG	Mix-breed	M	10.1	Degenerative h.	107	Yes	59	no
**2**	TG	Maltese	MN	13.3	CCHBD	1693	No	409	no
**3**	TG	CKCS	M	9.9	CCHBD	299	No	118	no
**4**	TG	Cocker Spaniel	M	7.8	CCHBD	45	No	33	no
**5**	TG	Mix-breed	FN	2.6	Chronic hepatitis	465	No	329	no
**6**	TG	Golden Retriever	M	8.8	CCHBD	25	Yes	34	no
**7**	TG	Dachshund	F	14.7	CCHBD	38	No	44	no
**8**	TG	Shih Tzu	F	14.6	CCHBD	37	No	45	no
**9**	TG	French bulldog	FN	3	Chronic hepatitis	683	No	273	no
**10**	TG	Mix-breed	M	13.4	CCHBD	51	Yes	56	no
**11**	TG	Yorkshire	M	11.7	CCHBD	713	No	943	no
**12**	TG	Mix-breed	FN	10.2	Chronic hepatitis	46	Yes	956	no
**13**	TG	Toy poodle	F	13.5	CCHBD	30	No	27	no
**14**	TG	Mix-breed	FN	8.9	CCHBD	690	Yes	53	no
**15**	TG	Mix-breed	M	9.4	Chronic hepatitis	877	Yes	413	yes
**Median age (range)**			10 (2.6–14.7)						
**16**	CG	Mix-breed	M	7.6	CCHBD	61	No	52	no
**17**	CG	Mix-breed	FN	8.6	Chronic hepatitis	178	No	230	yes
**18**	CG	Mix-breed	MN	10.3	CCHBD	57	No	61	no
**19**	CG	English Setter	M	9.2	CCHBD	126	No	120	no
**20**	CG	Jack Russel Terrier	F	14.0	Degenerative h.	56	No	107	no
**21**	CG	WHWT	F	10.3	Chronic hepatitis	103	No	91	no
**22**	CG	Breton	F	7.6	Degenerative h.	48	No	68	no
**23**	CG	Bull terrier	MN	10.0	CCHBD	30	No	31	no
**24**	CG	CKCS	FN	8.8	Chronic hepatitis	52	No	72	no
**25**	CG	CKCS	FN	7.7	CCHBD	2652	No	41	no
**26**	CG	WHWT	F	10.7	Degenerative h.	54	Yes	57	no
**27**	CG	Labrador	M	9.6	CCHBD	311	Yes	350	no
**28**	CG	Toy poodle	F	5.7	CCHBD	204	Yes	122	yes
**29**	CG	Boxer	F	9.3	Degenerative h.	467	No	435	yes
**30**	CG	Mix-breed	FN	11.3	CCHBD	114	No	108	yes
**31**	CG	Yorkshire	FN	11.6	CCHBD	127	No	195	no
**Median age (range)**			9.4 (5.7–14)						

Legend: CKCS: Cavalier King Charles Spaniel; WHWT; White Highland White Terrier; Degenerative h.: degenerative; CCHBD: chronic cholestatic hepatobiliary disease; F: female; FN: female neutered; M: male; MN: male neutered; GI signs: gastrointestinal clinical signs; ALT; alanine transferase.

**Table 3:** Descriptive statistics and differences in serum hepatic enzymes, total bilirubin (Tot Bil), cholesterol (Chol), triglycerides (Trig), total protein (TP), and albumin (Alb) expressed as median and range in CHBD dogs considering TG (treatment group) and CG (control group) at T0. Applied statistical test: Mann–Whitney U test.

Biochemical Parameter	Treatment Group	Control Group	Reference Range	*p*-Value
**ALP** (U/L)	512 (86–8907)	576 (74–8839)	45–250	0.82
**GGT** (U/L)	3.9 (1.1–375)	5.4 (2.2–70)	2–11	0.59
**AST** (U/L)	37 (18–324)	35 (20–1493)	15–40	0.94
**ALT** (U/L)	107 (25–1697)	108 (30–2652)	20–70	0.90
**Tot Bil** (mg/dL)	0.22 (0.1–1.55)	0.2 (0.07–6.55)	0.07–0.3	0.27
**TP** (g/dL)	6.9 (4.4–9.1)	7 (5.2–9.8)	5.8–7.8	0.54
**Alb** (g/dL)	4 (2.7–4.6)	3.9 (2.8–5.4)	2.6–4.1	0.91
**Chol** (mg/dL)	297 (111–673)	318 (90–677)	120–280	0.57
**Trig** (mg/dL)	130 (46–1475)	114 (45–654)	25–90	0.91

**Table 4:** Descriptive statistics (medians and range) of fecal alpha diversity and number of species (OTU) in TG (treatment group) and CG (control group) at T0 and T1 timepoints.

	Treatment Group		Control Group	
Timepoint	T0	T1	T0	T2
**Alpha diversity**	2.407 (0.831−3.163)	2.533 (1.501−3.197)	16,000 (9000−32,000)	39,000 (26,000−48,000)
**N species**	15,000 (11,000−19,000)	16,000 (13,000−23,000)	2.200 (0.839−3.491)	3.292 (2.207−4.098)

**Table 5:** Data expressed as median and range in CHBD dogs considering group TG and CG.

	Treatment Group			Control Group		
	T0	T1	*p*-Value	T0	T1	*p*-Value
**CDCA**	162.9 (56.6–705.3)	396 (75.4–999.7)	0.06	391 (12.9–861.1)	458.3 (47.6–1390)	0.27
**UDCA**	197.1 (32.5–689.3)	114.8 (18.7–669.6)	0.85	277.3 (112.7–1734)	246.1 (5.8–1332)	0.81
**CA**	377.6 (9.1–1305)	245.3 (18.9–1513)	0.36	239.3 (22.5–1303)	100.7 (16.6–726.5)	0.34
**DCA**	238 (7.5–2351)	412.1 (7.15–1578)	0.24	407.4 (9.05–1928)	283.4 (3.5–1416)	0.48
**LCA**	538.4 (48.4–1209)	458.7 (30.39–1392)	0.81	605.5 (155.8–1195)	372 (23.3–1077)	0.22
**Total Primary**	656.6 (69.3–1472)	665.4 (331–1943)	0.09	512.8 (146.4–1823)	655.1 (88.7–2104)	0.98
**Total Secondary**	910.1 (168.5–3705)	1011 (139.7–3031)	0.48	1198 (379.7–3456)	948.2 (33.7–2820)	0.46
**Ratio P/S**	0.35 (0.06–2.3)	0.7 (0.2–2.4)	0.94	0.5515 (0.07–1.56)	0.598 (0.2–2.634162)	0.7

Legend: Chenodeoxycholic acid (CDCA), Cholic acid (CA), Ursodeoxycholic acid (UDCA), Deoxycholic acid (DCA), Lithocholic acid (LCA), Total Primary (CDCA + CA), Total Secondary (UDCA+DCA+LCA), Ratio P/S (Total Primary/Total Secondary).

## Data Availability

The complete data set is available upon reasonable request.
